# Genome-wide identification of *CBF* genes and their responses to cold acclimation in *Taraxacum kok-saghyz*

**DOI:** 10.7717/peerj.13429

**Published:** 2022-05-12

**Authors:** Haifeng Zhang, Yongyong Gong, Peilin Sun, Sixue Chen, Chunquan Ma

**Affiliations:** 1 Engineering Research Center of Agricultural Microbiology Technology, Ministry of Education, Heilongjiang University, Harbin, China; 2 Key Laboratory of Molecular Biology, College of Heilongjiang Province, School of Life Sciences, Heilongjiang University, Harbin, China; 3 Key Laboratory of Nuclear Technology Application, Heilongjiang Institute of Atomic Energy, Harbin, China; 4 Proteomics and Mass Spectrometry, Interdisciplinary Center for Biotechnology Research, University of Florida, Gainesville, FL, USA; 5 Department of Biology, Genetics Institute, Plant Molecular and Cellular Biology Program, University of Florida, Gainesville, FL, USA

**Keywords:** *CBF*, *Taraxacum kok-saghyz*, Genome-wide analysis, Bioinformatics, Gene expression, Cold acclimation

## Abstract

C-repeat binding factors (CBFs) are transcription factors that are known to play important roles in plant cold acclimation. They are highly conserved in most higher plants. *Taraxacum kok-saghyz* (TKS) is an herb native to China and Kazakhstan and is well-known for its production of rubber silk with industrial and economic value. To understand cold acclimation mechanisms, we conducted a genome-wide discovery of the *CBF* family genes in TKS and revealed ten *CBF* genes. A bioinformatic analysis of the *CBF* genes was carried out to analyze the phylogenetic relationship, protein conservative motifs, protein physicochemical properties, gene structure, promoter *cis*-acting elements, and the gene expression patterns under cold acclimation and control conditions. It was found that most of these genes were highly responsive at the late stage of cold acclimation, indicating that they play important roles in the cold acclimation processes of TKS. This study provides a theoretical basis for the study of the molecular functions of the *CBF* gene family in TKS, and a useful guidance for the genetic improvement of the cold tolerance traits of TKS and other plants, including crops.

## Introduction

Low temperature is one of the severe abiotic stresses which negatively affects plant growth and development. To respond to and tolerate the low temperature stress, plants have evolved complex and efficient molecular regulatory mechanisms (Zeng et al., 2021; Zhang et al., 2022). Many plants increase their freezing stress tolerance upon exposure to low but nonfreezing temperatures, a phenomenon known as cold acclimation (Thomashow, 1999). Cold acclimation is a self-protection response to low temperature, and has a positive impact on the improvement of low temperature adaptation of crops. It is a complex process involving many molecular, biochemical, and physiological changes (John et al., 2016). It has been reported that cold acclimated *Sonneratia apetala* seedlings have lower relative electrolyte leakage and malondialdehyde (MDA) content than non-acclimated seedlings (Shen et al., 2021). Furthermore, it has been shown that cold acclimated *Hordeum vulgare* (barley) plants have higher cold tolerance and photosynthesis rates than non-acclimated control plants (Jurczyk et al., 2018).

In recent years, the molecular mechanisms underlying cold acclimation have been widely studied, and efforts have been made to explore the key regulatory factors of this complex network (Liu et al., 2019). For example, an inducer of CBF expression 1 (ICE1)–C-repeat (CRT)-binding factors (CBF)–cold responsive (COR) signaling pathway in plant cold stress response has been recently reviewed (Hwarari et al., 2022). In *Arabidopsis thaliana*, cells can sense cold temperature through calcium/calmodulin-regulated receptor-like kinases (CRLKs) at the plasma membrane. Following cold sensing, an increase in cytosolic Ca^2+^ triggers the expression of *CBFs*, which bind to C-repeat/dehydration-responsive motif (CRT/DRE, G/ACCGAC) in the downstream COR gene promoter region, inducing the expression of *COR* (Gilmour et al., 1998). AP2 domain in CBFs directly binds to CRT/DRE *cis*-element in cold-regulated *COR* promoter (Tang et al., 2020). In *A. thaliana*, some CBF target genes, such as *COR15A* and *COR78*, are particularly relevant to cold acclimation. For example, overexpression of *COR15* significantly enhanced freezing resistance (Rocco et al., 2013). The CBFs are to some extent conserved in the plant domain. They belong to a dehydration responsive element binding protein (DREB) subfamily A-1 subgroup in the AP2/ERF transcription factor family (Mizoi, Shinozaki & Yamaguchi-Shinozaki, 2012). The CBFs are plant specific transcription factors; their protein structures contain a highly conserved AP2/EREBP DNA binding domain composed of about 60 amino acids (Lata & Prasad, 2011). *Genome of A. thaliana contains six CBF genes AtCBF1*, *AtCBF2*, *AtCBF3*, *AtCBF4*, *AtCBF5*, and *AtCBF6.* Each of them acts in a different pathway and is differentially expressed under stress conditions (Wang et al., 2014). For example, *AtCBF4* is not induced by cold, but its overexpression in *A. thaliana* can enhance plant tolerance to frost and drought (Haake et al., 2002). Emerging studies in Arabidopsis have explored the transcriptional network of cold acclimation pathways. Compared with the wild type, the freezing tolerance of *A. thaliana CBF1* and *CBF3* knock-out mutants decreased by about 60% (Novillo, Medina & Salinas, 2007). *CBF1/2/3* triple mutants had little freezing-sensitive phenotype under normal growth conditions, but showed strong freezing-sensitive phenotype after cold acclimation (Park et al., 2015; Zhao et al., 2016). Through RNA-Seq analysis of *CBF1/2/3* triple mutants, it was found that mutation of *CBFs* affects 10%∼20% of the *COR* gene expression in the whole transcriptome, indicating other factors important for cold tolerance may function downstream of CBFs (Jia et al., 2016). CBFs clearly play a key role in cold acclimation (Liu et al., 2019). Many studies have demonstrated that CBFs are involved in the transcriptional regulation in *Liriodendron chinense* (Guan et al., 2021), *Triticum aestivum* (Zan et al., 2020), *Lactuca sativa* (Park, Shi & Mou, 2020), *Brassica napus* (Ghorbani et al., 2020), *Brassica oleracea* (Thamilarasan et al., 2014), *Betula* (Lv et al., 2020), soybean (Yamasaki & Randall, 2016), and strawberry (Fattash et al., 2021). Silencing of the *CBF* genes significantly reduced the frost tolerance of *Betula* and *Brachypodium distachyon* (Hao et al., 2017).

Natural rubber (NR) is an important industrial material related to the national economy and people’s livelihood. Rubber grass (*Taraxacum kok-saghyz*, TKS) is a perennial herb of the Compositae family commonly referred to as the Kazakh or Russian dandelion. It is native to Tianshan Mountains of China and Kazakhstan (Kirschner et al., 2013). It is well-known for its rubber production together with the rubber tree and the silver rubber Chrysanthemum. Dry roots of TKS contain rubber silk, which has industrial and economic value. TKS are characterized by strong environmental and cold temperature adaptability; thus, they thrive in cold areas with strong growth and reproductive ability. Therefore, TKS is a great plant for studying the functions of *CBF* genes in cold tolerance and in improving rubber production. TKS contains large amounts of *cis*-1,4-polyisoprene in its enlarged roots, which is an alternative plant source of NR (Xie et al., 2019). The amount of NR in the TKS roots is similar to that in the rubber tree *Hevea brasiliensis* (Kirschner et al., 2013). Therefore, TKS is a model plant for studying NR biosynthesis as it possess a relatively small genome and a fast growth-cycle (Schmidt et al., 2010). Its genome is relatively simple (1.29 Gb), containing 46,731 protein-coding genes (Lin et al., 2018). With the completion of TKS genome sequencing, study of key functional genes at the genome-wide level as well as cloning and expression analysis of resistance-associated genes has become possible. It is valuable to be able to guide the applied research of stress-related genes in TKS and other important crops. Cold stress is a major factor limiting the planting of TKS. Cold acclimation can advance the germination period of TKS in the spring and delay the withering period in autumn so as to improve rubber production by prolonging the growth period. Therefore, it is of great practical significance to study the mechanisms underlying the cold acclimation of TKS. At present, the research on TKS mainly focuses on rubber extraction, structural characterization, variety screening, and rubber synthesis-related genes. There is a lack of systematic research on gene families at the whole genome scale.

This study is aimed to understand the structure and function of the CBF family in TKS. With the continuous advancement of genomic research, gene family analysis at the whole genome level has become a reality (Wang et al., 2019a; Wang et al., 2019b). The acquisition of TKS genomic data provides a basis for the analysis of its gene family (Lin et al., 2018), which facilitates further understanding of gene functions and regulatory mechanisms. Here we studied the TKS *CBF* gene family, including gene structure, *cis*-acting elements, conserved motifs, phylogenetic context, and gene expression profiles during cold acclimation. These results have contributed to a deeper understanding of the *CBF* gene structure and function in plant cold stress response and cold acclimation.

## Materials & Methods

### Plant materials, cold acclimation treatment analysis

**Figure 1:**
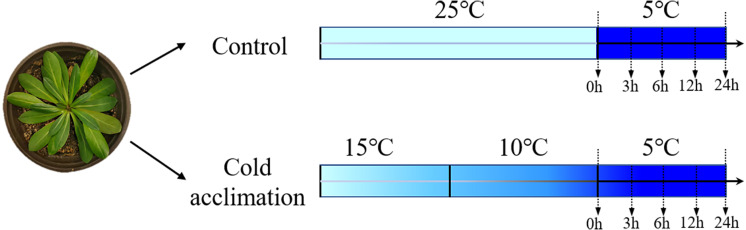
Experimental design of cold acclimation of seedlings. Schematic diagram of *Taraxacum kok-saghyz* seedlings subjected to different temperature treatments. The control seedlings were grown at 25 °C followed by cold treatment at 5 °C for 0, 3, 6, 12 and 24 h. Cold-acclimation was conducted at 15 °C and 10 °C, each for one day, followed by cold treatment at 5 °C for 0, 3, 6, 12 and 24 h.

TKS seeds were provided by the College of Life Sciences, Heilongjiang University, China. They were sown in pots (inner diameter 14 cm, height 15 cm) with seedling vermiculite soil, and grown at room temperature 25 °C under a light intensity of 100 µmol m^−2^ sec^−1^. At four-pair-leaf growth stage, the seedlings were selected and divided into two groups: (1) control: non-acclimated seedlings grown at room temperature 25 °C, followed by at low temperature 5 °C as cold treatment, and (2) cold acclimation: grown at 15 °C for 1 day, followed by a second day at 10 °C prior to a cold treatment at 5 °C (Fig. 1). Leaves were collected at 0, 3, 6, 12, and 24 h for each group of treatments. They were frozen immediately in liquid nitrogen for RNA extraction according to Shen et al. (2021). For each sample, three biological replicates and three technical replicates were conducted.

### Identification of *CBF* family genes

*A. thaliana CBF* genome sequences were downloaded from TAIR (http://www.arabidopsis.org). The translated CBF protein sequences were used for local BLAST analysis in the TKS database (Lin et al., 2018), and the corresponding CBF homologous genes were screened with an e-value threshold of <10^−5^. In addition, the presence of the AP2-domain was examined using the hmmscan function of HMMER3.0 (http://hmmer.org) (Potter et al., 2018) with the AP2 domain profile (Pfam accession, PF00847). Pfam scan (http://pfam.xfam.org/) verifies whether the candidate protein contains the AP2 domain. Multiple sequence alignment with Clustal Omega program was conducted to determine whether there are PKKPAGR and DSAWR sequences before and after the AP2 domain. The protein sequences with these two characteristics were assigned to the TKS CBF family. TBtools software was used to analyze conserved amino acid residues. Through the website analysis tool ProtParam (http://web.expasy.org/protparam/), ExPASy (https://web.expasy.org/protscale/) (Artimo et al., 2012) and PSORT (http://psort.HGC.JP/), the basic physical and chemical properties of proteins, such as molecular weight (Da), isoelectric point (pI), hydrophilicity, and subcellular localization were analyzed.

### Phylogenetic analysis, gene structure, conserved motifs, and chromosomal mapping analysis of the TKS *CBF* genes

The amino acid sequence data of *A. thaliana*, *Zea mays*, and *Oryza sativa* were downloaded from TAIR and Phytozome (https://phytozome.jgi.doe.gov/pz/portal.html). The *Lactuca sativa* protein sequences were downloaded from Genome Database (https://hardwoodgenomics.org). Multiple sequence alignments of the identified CBF amino acid sequences from the five species (TKS, *Z. mays*, *L. sativa*, *A. thaliana*, and *O. sativa*) were done using Clustal X2.1 (http://www.clustal.org) (Larkin et al., 2007) and MEGA 7.0 (Kumar, Stecher & Tamura, 2016). A phylogenetic tree was constructed using a neighbor-joining (NJ) method with the default parameters. GFF files with *TkCBF* chromosome location information were extracted from the TKS genome annotation files based on their starting and ending positions in the chromosomes. Using mapinspect software (https://mapinspect.software.informer.com/), the chromosome locations of *TkCBFs* were analyzed according to the genome annotation information. Gene intron-extron structure information was collected from the genome annotations of TKS from TKS databases. Then, the exon–intron structures for TKS CBF genes were checked and measured by analysis the sequences (Liu et al., 2013). The *TkCBF* gene structure was mapped using the TBtools software. The full-length protein sequences were analyzed using the MEME online program (https://meme-suite.org/meme/) (Bailey et al., 2015) to determine the distribution patterns of protein conserved motifs.

### Promoter *Cis-*acting elements analysis of the *TkCBF* Genes

Based on the genome annotation information provided by the TKS database, a 2,000 bp sequence upstream of the transcription start site of the *TkCBF* genes was extracted and submitted to Plant CARE (http://bioinformatics.psb.ugent.be/webtools/plantcare/html/) for promoter prediction. GSDS (http://gsds.gao-lab.org/) (Hu et al., 2015) online software was used to draw a distribution map of *cis*-regulatory elements.

### Expression analysis by qRT-PCR

Total RNA was extracted from TKS leaves without cold acclimation at 15 °C and 10 °C (25 °C, 5 °C) and cold acclimation at 15 °C and 10 °C (Fig. 1) using the Trizol method. Total RNA (1 µg) was used for reverse transcription. The first strand cDNAs for qRT-PCR were synthesized using PrimeScript™RT Master Mix (TaKaRa, Beijing, China). qRT-PCR was performed with PowerUp™SYBR™Green Master Mix (Thermo, Beijing, China), and 18S rRNA was used as the internal reference gene in this experiment. Premier5 was used to design the specific primers for the *TkCBF* genes (Table S1). Melting curves were acquired to determine primer specificity. Each PCR was performed using three biological duplicates and three technical replicates. Relative gene expression levels were calculated using the 2^−ΔΔCt^ and ANOVA.

## Results

### Genome-wide identification of *TkCBF* genes

Sequences of *CBF* genes in the *A. thaliana* genome were retrieved from TAIR for local blast identification of *TkCBF* genes. A total of 24 *TkCBF* candidate genes were identified by blastp (*E*-value < 10^−5^). After querying the consensus protein sequence of AP2/ERF by hidden Markov model (HMM), Pfam scan (http://pfam.xfam.org/) verifies whether the candidate protein contains the AP2 domain. Multiple sequence alignment with Clustal Omega program was used to determine whether there are PKKPAGR and DSAWR sequences before and after the AP2 domain. A total of 10 *TkCBF* genes were identified (Table S2) and named as *TkCBF1-TkCBF10* (Table 1). The physicochemical parameters of TKS CBF proteins were analyzed by ExPASy online tool. The theoretical PI values ranged from 4.72 to 6.23; this result indicates that the TkCBF proteins are weakly acidic. The molecular weight (Mw), amino acids (aa), scaffold position (Sp), and open reading frame (ORF) length are summarized in Table 1.

**Table 1:** Information on the *CBF* family genes identified in the TKS genome.

Gene name	Scaffold position	Gene ID	PI	Mw (kD)	ORF (bp)	Subcellular localization
TkCBF1	utg2547	evm.TU.utg2547.14	5.27	24049.19	645	nucleus
TkCBF2	utg19621	evm.TU.utg19621.11	4.72	23021.47	639	nucleus
TkCBF3	utg4527	evm.TU.utg4527.1	6.04	25050.64	663	nucleus
TkCBF4	utg14372	evm.TU.utg14372.5	4.89	23145.74	618	nucleus
TkCBF5	utg4653	evm.TU.utg4653.25	5.55	24972.55	666	nucleus
TkCBF6	utg4653	evm.TU.utg4653.20	5.59	25166.73	666	nucleus
TkCBF7	utg19621	evm.TU.utg19621.3	5.07	24707.82	672	nucleus
TkCBF8	utg19621	evm.TU.utg19621.4	5.22	24196.81	651	nucleus
TkCBF9	utg16021	evm.TU.utg16021.12	5.36	24295.95	651	nucleus
TkCBF10	utg16021	evm.TU.utg16021.13	6.23	24504.21	663	nucleus

### Analysis of protein characteristics and chromosome location of *TkCBFs*

**Figure 2:**
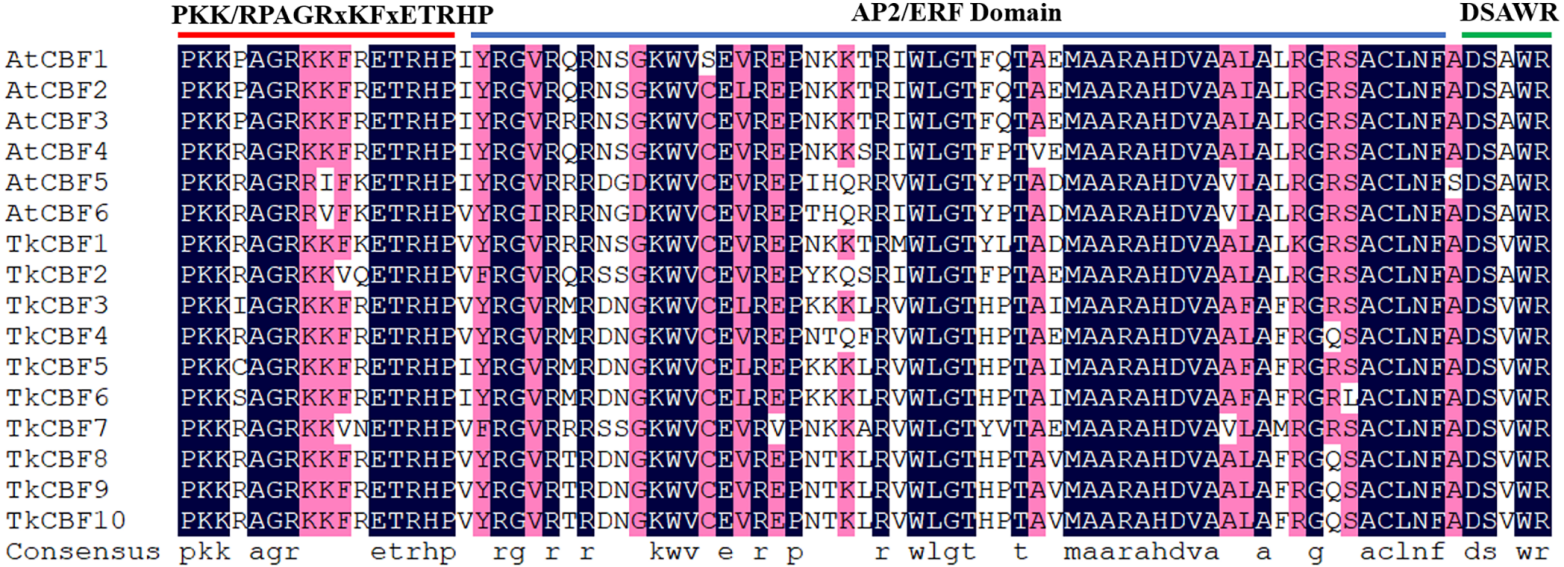
Multiple sequence alignment of the *Taraxacum kok-saghyz* CBF family proteins and the *A. thaliana* CBFs. The conserved sequences of the three conserved domains are shown at the top. The red line represents the upstream conserved domain of AP2, and the green line indicates the downstream conserved domain of AP2. Blue amino acids represent complete conservation, red represents 75% conserved amino acids, white highlights non-conservative amino acids.

The phylogeny of a species can be reflected by its homology, and therefore can be used to help annotate newly sequenced genes based on known genes. To gain a better understanding of the similarity and dissimilarity of motifs in different TkCBF proteins, we performed multiple sequence alignment and motif distribution analysis. Sequence alignment analysis of the 10 TKS CBFs and six *A. thaliana* CBF proteins revealed that the AP2/ERF domain of TkCBF proteins has high homology with the characteristic motif of the CBF family. All the identified TkCBFs contained the previously reported CBF-specific conserved domain PKK/RPAGRxKFxETRHP-AP2/ERFdomain-DSAWR motif (Canella et al., 2010) (Fig. 2).

**Figure 3:**
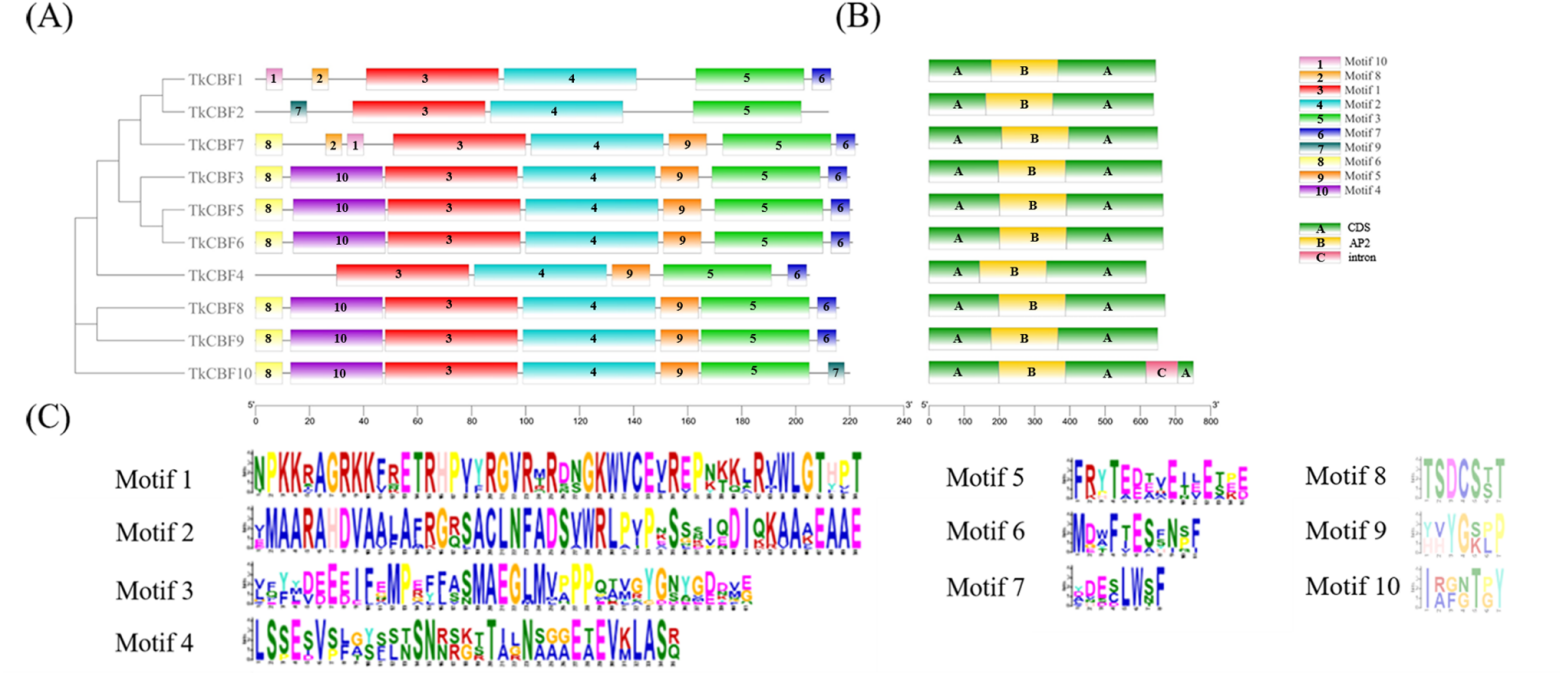
Conserved motifs and structure of TkCBFs. (A) A Phylogenetic relationships (left) and conserved motifs (right); (B) gene structure of the 10 TKS *CBF* genes. (C) Important Motif logos with the amino acid composition of each motif. Different colored boxes represent different themes and their positions in each TkCBF sequence. CDS refers to the coding sequence of a gene.

Further analysis of the distribution of the TkCBF conserved domains showed 10 conserved motifs predicted by MEME. The 10 motifs found were named Motif 1–Motif 10 (Fig. 3A). Each TkCBF protein has its specific conserved motifs. Since the conserved motifs are highly similar, proteins with the same motifs may be functionally similar. Motif analysis found that all CBFs contained motifs 1, 2, and 3 (Fig. 3A). Combined with the results of multiple sequence alignment (Fig. 2), motif 2 was a typical AP2 domain, and was highly conserved among the TkCBF family proteins. Please note that there are no non-coding sequences, which is consistent with the CBF study of the tea tree (Hu et al., 2020). The conserved motifs may play special functions. The CBFs in TKS with close evolutionary relationships usually have similar structure and number of conserved motifs, implying they may have similar functions.

**Figure 4:**
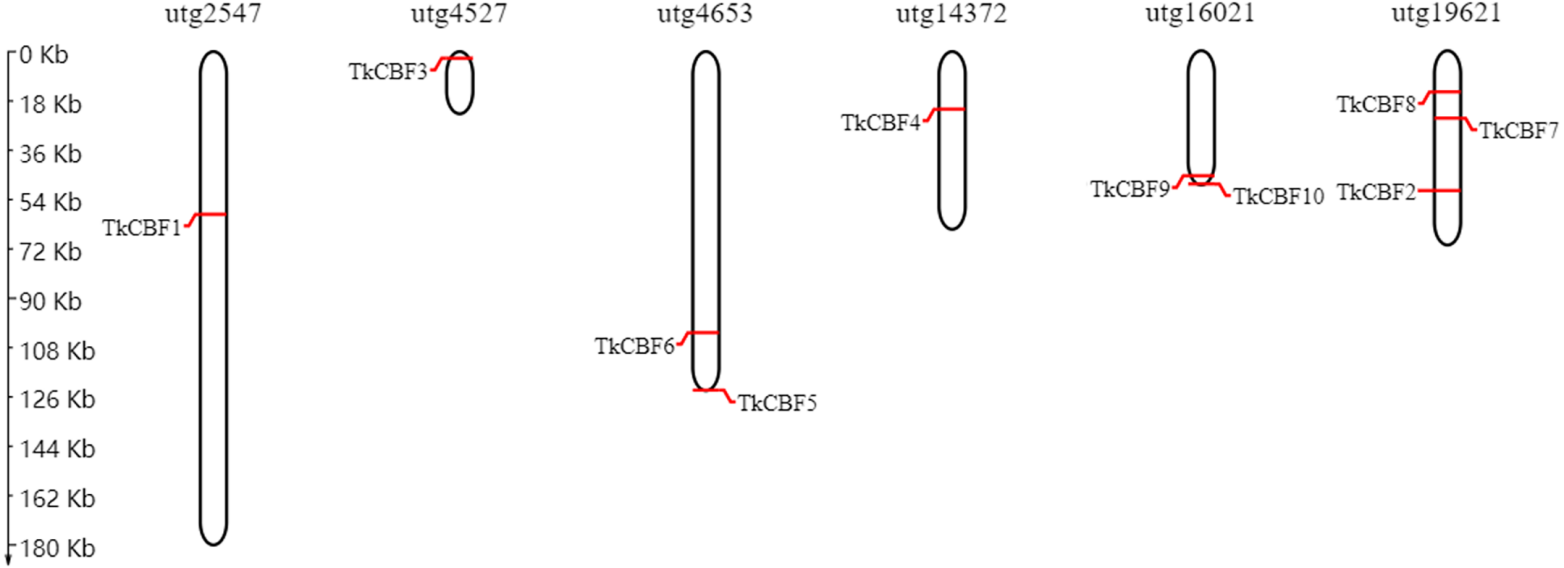
Chromosomal location of the 10 *Taraxacum kok-saghyz CBF* genes. The genes are located over the six linkage groups: utg2547, utg4527, utg4653, utg14372, utg16021 and utg19621.

The chromosomal map constructed revealed that the 10 *TkCBF* genes were distributed on six independent genome assembly scaffold fragments (Fig. 4). The scaffold utg19621 contained the largest number of *TkCBFs* (three genes), and two genes on both scaffold utg4653 and utg16021. Other scaffolds each contain one gene, and the distribution of genes in the different scaffolds is inconsistent (Fig. 4) due in large part to differences in the structure and size of the chromosomes.

### Phylogenetic and gene structure analyses of the TKS *CBF* gene family

**Figure 5:**
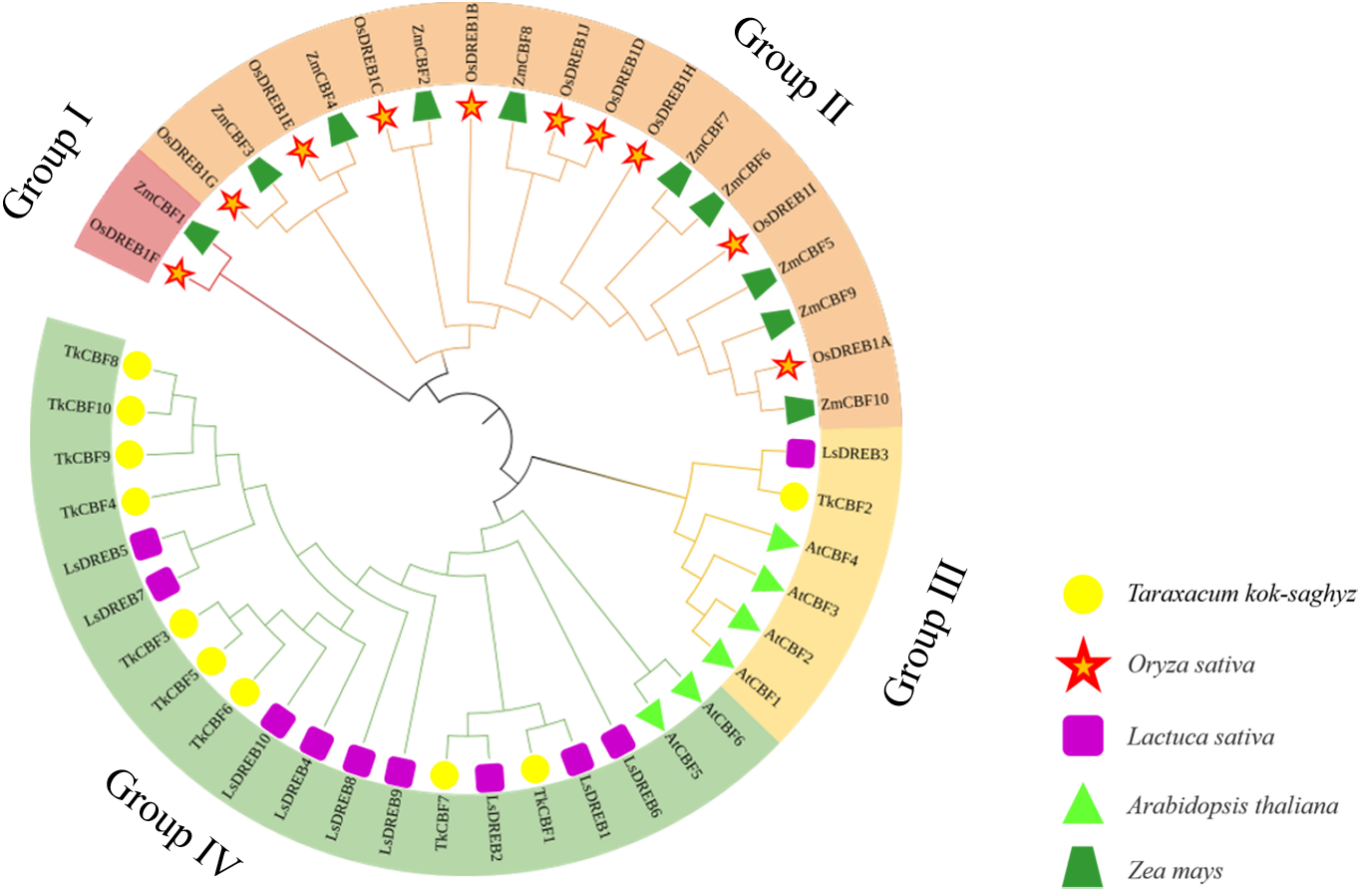
Phylogenetic analysis of CBF proteins in five species. The circular unrooted tree was generated by NJ method with 1,000 bootstrap replicates. Different phylogenetic groups are represented by different colors.

To study the phylogenetic relationship of the CBFs in TKS and other plants, the members of CBF families from TKS, *A. thaliana*, *O. sativa, Z. mays*, and *L. sativa* were collected to construct the phylogenetic tree. Forty-six CBF members were divided into four groups (Fig. 5). Group I and Group II contained the CBF genes of rice and maize, but does not include the *A. thaliana*, *L. sativa*, and *TKS CBF* genes. It is speculated that there is a species differentiation in their origin. Group III and Group IV contained *L. sativa* DREB genes and TKS *CBF* genes, indicating that these genes co-exist in the same family and Asteraceae. This result implied the difference in evolution between Groups I–II and Groups III–IV. The result clearly showed close relationships between TKS and *L. sativa CBF* genes, since the genes from these two species were closely clustered in different groups and subgroups of the phylogenetic tree. The 10 *TkCBFs* in Groups III and IV were homologous to *AtCBFs*, *LsDREBs*, and *TkCBFs* were closely related to *A. thaliana* and *L. sativa*, indicating the expression and functional diversity of *CBF* family members. An important factor that determines the level of gene expression and function is the structure of the gene. The gene structure analysis of *TkCBFs* revealed that all members of the family contain AP2 structural domains, which are strongly conserved. The gene structure of *TkCBFs* is consistent with the evolutionary results of its members (Fig. 3B). Among the 10 *TkCBF* genes in TKS, only *TkCBF10* contained one intron. The intron phase was found to be a type 1 intron by sequence analysis (Fig. 3B), and the other 9 *TkCBFs* were all single-exon structures, presumably all members of this family were intron deletion genes except *TkCBF10*. The loss of introns may shorten the time required for gene transcription to translation, therefore enabling fast gene expression, and production of functional proteins in response to changes in the plant or the environment (Shu et al., 2015).

### *Cis-*acting elements analysis of the promoter region of *TkCBFs*

**Figure 6:**
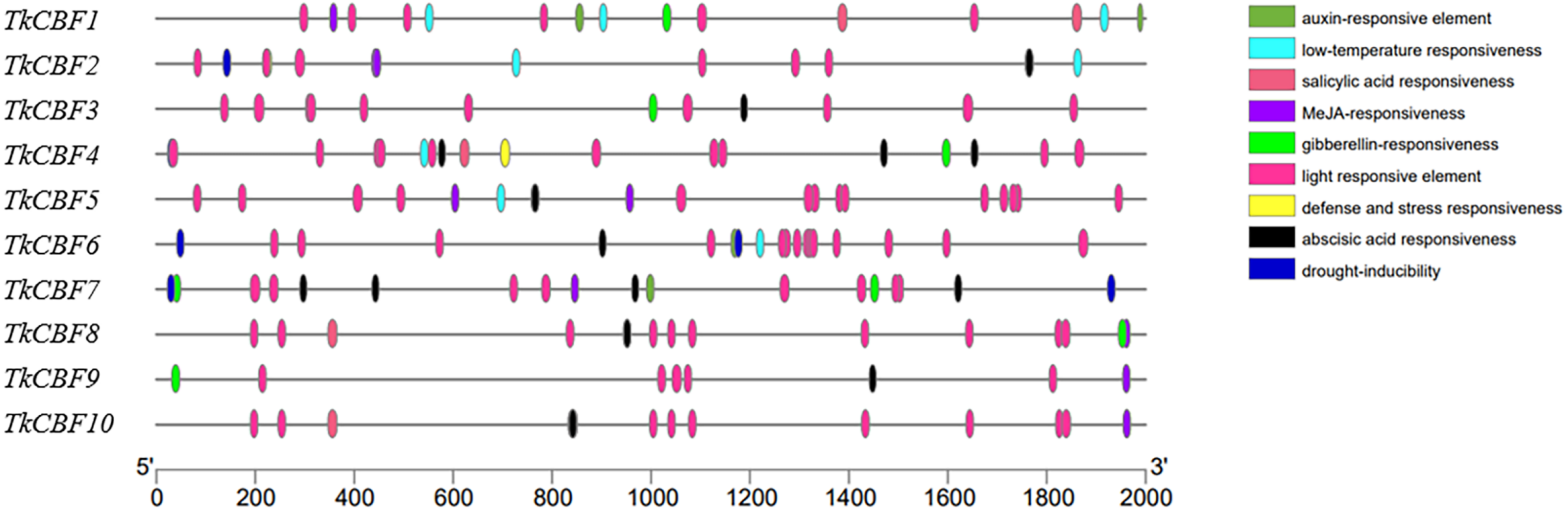
The *cis*-acting elements of the promoter regions of *TkCBFs*. The 2,000 bp nucleotide sequence upstream of the transcription start site was used as a promoter *cis*-acting element for analysis. Different colored ovals represent different elements and their positions in each *TkCBF* promoter.

Promoter analysis can help further explore the regulatory mechanisms of *TkCBF* family members. As shown in Fig. 6, *cis*-acting elements were predicted in the promoters of the 10 *TkCBF* genes. Ten types of *cis*-acting elements relevant to stresses (light, drought, and low-temperature) and phytohormone responses (abscisic acid, auxin, salicylic acid, and methyljasmonate) were discovered in the promoters of the *TkCBF* genes. Interestingly, the light-responsive elements accounted for the largest portion of all the identified *cis*-elements (Table S3). Among the numerous cis-acting elements, we also identified several MYC motifs, which are binding sites for ICE1 (Table S3). ICE1 is mainly involved in the regulation of the expression levels of *CBFs* under cold stress, further supporting that TkCBFs may be involved in the ICE1-CBF-COR cold signaling pathway. The results suggest that the expression of *TkCBF* genes might be associated with different environmental factors, and could potentially play important roles in plant growth and response to harsh environmental conditions. The *cis*-element data can springboard further hypothesis-testing studies on the expression characteristics and functions of the ten *TkCBF* genes.

### Expression analysis of *TkCBF* genes under cold acclimation

**Figure 7:**
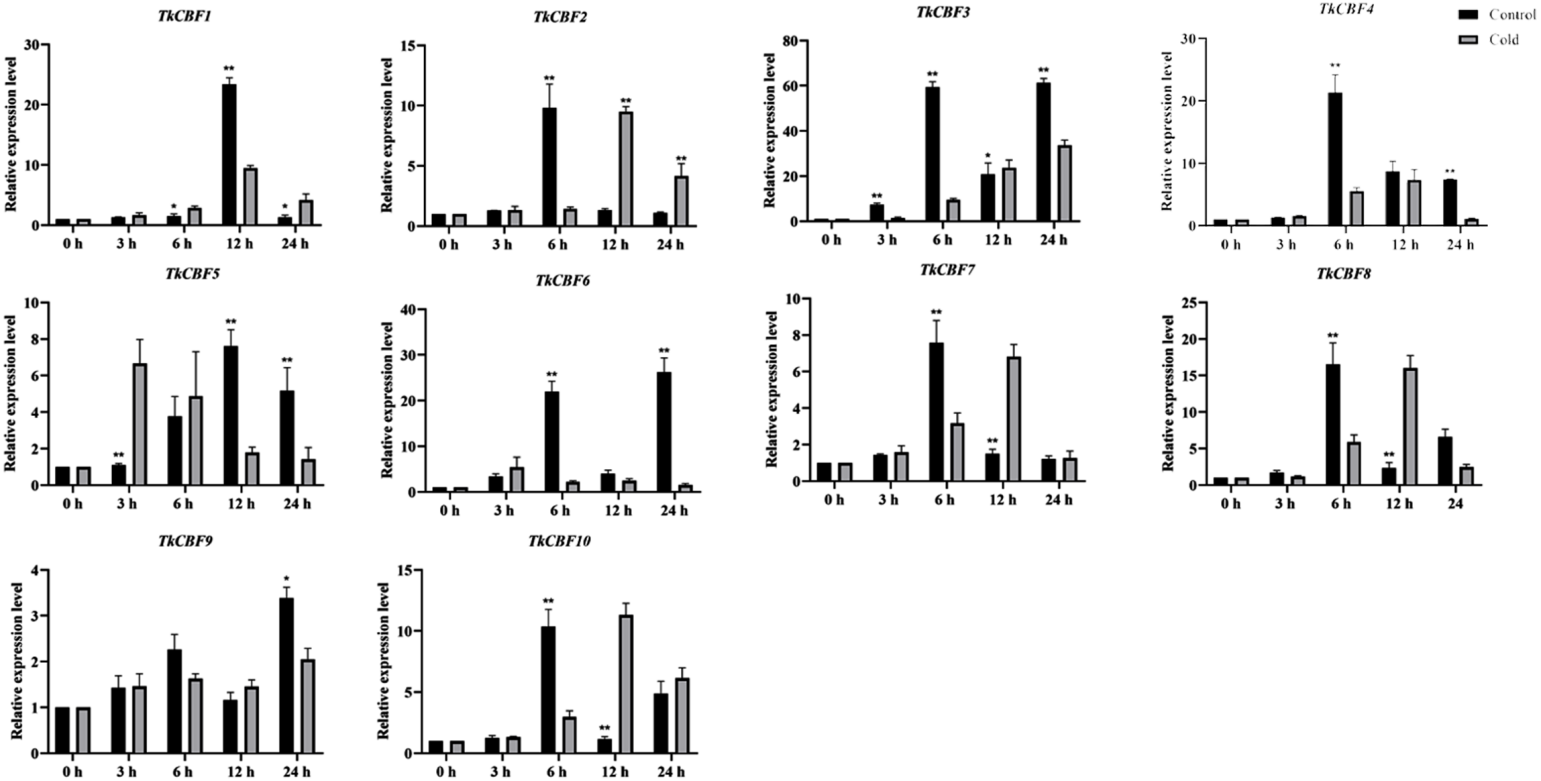
Expression profiles of *TkCBF* genes in response to cold acclimated and non-acclimated (control) conditions. qRT-PCR expression levels of the 10 *CBF* genes were determined using the 2^−ΔΔCt^ method with *18S RNA* as the internal standard for normalization. The results are average with standard deviation of three biological replicates.

Here we investigate the expression profiles of the 10 *TkCBF* genes in the leaves of TKS under cold acclimation and control conditions (Fig. 7). Although the 10 *TkCBF* genes showed similar expression patterns in response to cold acclimation, they exhibited differential timings and altitudes. For example, the expression of *TkCBF3*, *TkCBF5*, and *TkCBF6* showed an increase followed by a decrease in the trend and reached the highest expression level within 24 h. *TkCBF1*, *TkCBF2*, *TkCBF4*, *TkCBF7,* and *TkCBF8* showed a similar upward and then downward trend, however, peak expression was reached within 6 h or 9 h, implying that the *TkCBF1*, *TkCBF2*, *TkCBF4*, *TkCBF7*, and *TkCBF8* may be more sensitive to cold signal. Similarly, we also carried out real-time PCR of TKS leaves under non-acclimation/control condition. The expression of these 10 genes was also up-regulated, but the peak time was significantly later than those of cold-acclimated samples (Fig. 7). This finding is consistent with the results from *A. thaliana* (Gilmour Fowler & Thomashow, 2004; Lin et al., 2008).

## Discussion

CBF is a key transcription factor involved in plant cold acclimation (Shi, Ding & Yang, 2018), When plants are subjected to low temperature stress, CBF transcription factors are activated to regulate about 12% of the low temperature responsive transcriptome (Sun et al., 2014). *CBF* genes were discovered and explored in the reference plant *A. thaliana* (Thomashow, 2010). Ever since, *CBF* genes and their homologs have been increasingly studied to improve cold tolerance in other plants (Gehan et al., 2015; Francia et al., 2016). However, CBFs have been rarely reported in TKS, and their biological functions remain unclear. In this study, we conducted a genome-wide search for the CBF orthologs in TKS, and identified a total of 10 *CBF* genes. Compared with the reference species *A. thaliana*, TKS has more CBFs. This may be due to the slightly larger TKS genome, and its adaptation to adverse environmental conditions.

The physicochemical properties of the CBF proteins revealed that the theoretical isoelectric points of TkCBF proteins are in the similar range as those from *Punica granatum* and *Liriodendron chinense* (Guan et al., 2021). The predicted subcellular localization indicate that the TkCBFs are localized in the nucleus for biological roles as transcription factors. The results of the phylogenetic tree indicate that the TkCBFs are closely related to those in lettuce, which makes sense due to the fact that both species belong to the Asteraceae family.

Analyses of gene structure and conserved motif differences can help determine the evolutionary relationships of gene families (Zhou et al., 2020). The composition of introns and exons in the genes is important for their evolutionary analysis. The TKS genome contains only one exon and no intron except for the *TkCBF10* gene, which is consistent with the *A. thaliana AtCBF1/2/3* gene structure (Medina et al., 1999), and has a closer phylogenetic relationship. The gene structure analysis inferred that the *CBF* genes containing introns might have mutated during evolution. A total of 10 *TkCBF* family members were identified in this study, four more than the model species *A. thaliana*. This could be attributed to the difference in genome size and the degree of CBF gene amplification. CBFs within the same species have similar conserved motifs and gene structures. All the 10 *TkCBFs* genes encode proteins containing AP2 structural domains that are highly conserved, which is consistent with the results of *CBF* studies in *Camellia sinensis* (Wang et al., 2019a; Wang et al., 2019b). Intron-deficient genes can rapidly complete transcriptional processes under abiotic stress, enabling efficient plant response to stress signals (Giri et al., 2013). The *TkCBFs* are mostly intron-deficient genes, and it is hypothesized that they are more responsive to abiotic stresses. Most of the *CBF* gene family members in TKS consist of similar motifs, and the TkCBF proteins all have motifs 1, 2, and 3, suggesting that the functions of the CBF family proteins may be mediated by these three motifs. The similarity of most CBF proteins in gene structure and motif composition is consistent with the phylogenetic result of the CBF gene family. Compared with the *A. thaliana* cold-responsive gene *AtCBF1-3* (Fig. S1), which has extremely similar conserved motifs, we predict that all 10 *CBF* genes of TKS are likely to be cold responsive genes. These results suggest that the *CBF* gene family may have similar functions in the cold acclimation.

The promoter contains *cis*-acting elements located upstream of the transcriptional start, and they are at the center of gene transcriptional control. Analysis of promoter *cis*-acting elements showed that the promoters of all the 10 *TkCBF* genes have light-responsive signaling elements (Fig. 6). In *A. thaliana*, inhibition of phytochrome interaction factor (PIF) by shortening daylight hours resulted in up-regulation of *CBF* gene expression and improved freezing tolerance (Lee & Thomashow, 2012). Therefore, it is reasonable to hypothesize that *TkCBF* genes are involved in light signaling to regulate the growth and development processes. In addition, the expression of *CBFs* was affected by gibberellin (GA), jasmonic acid (JA), abscisic acid (ABA), and ethylene (ET). In addition, the present study screened the promoters of the 10 *TkCBF* genes for *cis*-acting elements responding to hormones such as ABA, GA, JA, auxin, and ET. This result will help us further elucidate the regulatory mechanisms of hormone signaling and cold acclimation in TKS.

Some reports showed that a triple mutant of *AtCBF1*, *AtCBF2*, and *AtCBF3* in *A. thaliana* was extremely sensitive to freezing stress compared with the wild type control (Zhao et al., 2016; Zhao et al., 2016). *AtCBF1*, *AtCBF2*, and *AtCBF3* were induced at 15 min under cold stress treatment, and their expression peaked at 2 h and then gradually decreased. (Gilmour et al., 1998). The expression patterns of *CBFs* in tomato and cotton were basically the same as that of *A. thaliana*. They all reached peak expression at 2 h. (Zhu et al., 2016). However, under cold treatment, the expression of most *TkCBFs* increased with the increasing treatment time, and showed a trend of increased expression, peaked at 6 h and then declined, a pattern similar to that of tea tree (Hu et al., 2020), *Prunus mume* (Zhao et al., 2018), and *Brassica rapa* L. (Jiang et al., 2011). In contrast to non-overwintering plants such as *A. thaliana*, tomato and cotton, TKS and tea tree usually experience a longer period of low-temperature chilling during the growing season. Therefore, the induction of *TkCBFs* during cold treatment takes longer time. It is also interesting to note that the expression pattern of *TkCBF2* is most similar to that of the cold responsive *AtCBFs*, consistent with their sequence similarity. Future experiments should focus on investigating the cold-responsive molecular functions of these TkCBFs (*e.g.*, their downstream target genes and pathways).

## Conclusions

CBF proteins play a prominent positive role in the process of cold acclimation, and they are highly conserved in higher plants. In our study, we identified 10 CBF members in TKS. A comprehensive phylogenetic analysis of CBF family members in *A. thaliana*, maize, rice, and lettuce showed evolutionary relationship. In addition, protein conserved domains, protein physical and chemical properties, gene structure, promoter *cis*-acting elements, and gene expression patterns under cold acclimation and non-acclimation conditions were studied. Most *CBF* genes in TKS seedlings after cold acclimation took longer to respond to cold signal than those without the acclimation. This research represents a theoretical basis for a comprehensive understanding of *TkCBF* gene family. The results will springboard further studies on the molecular functions of the TKS *CBF* gene family, and on future genetic improvement of cold tolerance of TKS and crops.

##  Supplemental Information

10.7717/peerj.13429/supp-1 Supplemental Information 1 Comparison of CBF motifs between Hevea brasiliensis and *A. thaliana*

10.7717/peerj.13429/supp-2 Supplemental Information 2 CBF1-10 Melt Curve

10.7717/peerj.13429/supp-3 Supplemental Information 3 Primer information of TkCBF family genes

10.7717/peerj.13429/supp-4 Supplemental Information 4 The sequences of CBF supplemental data

10.7717/peerj.13429/supp-5 Supplemental Information 5 Promoter supplemental data

10.7717/peerj.13429/supp-6 Supplemental Information 6 Gene expression

## Additional Information and Declarations

### Data Availability

The following information was supplied regarding data availability: The data is available at the Genome Warehouse: PRJCA000437.

## References

[ref-1] Artimo P, Jonnalagedda M, Arnold K, Baratin D, Csardi G, de Castro E, Duvaud S, Flegel V, Fortier A, Gasteiger E, Grosdidier A, Hernandez C, Ioannidis V, Kuznetsov D, Liechti R, Moretti S, Mostaguir K, Redaschi N, Rossier G, Xenarios I, Stockinger H (2012). ExPASy: SIB bioinformatics resource portal. Nucleic Acids Research.

[ref-2] Bailey TL, Johnson J, Grant CE, Noble WS (2015). The MEME suite. Nucleic Acids Research.

[ref-3] Canella D, Gilmour SJ, Kuhn LA, Thomashow MF (2010). DNA binding by the Arabidopsis CBF1 transcription factor requires the PKKP/RAGRxKFxETRHP signature sequence. Biochimica Et Biophysica Acta/General Subjects.

[ref-4] Fattash I, Deitch Z, Njah R, Osuagwu N, Mageney V, Wilson RC, Davik J, Alsheikh M, Randall S (2021). Accumulation dynamics of transcripts and proteins of cold-responsive genes in fragaria vesca genotypes of differing cold tolerance. International Journal of Molecular Sciences.

[ref-5] Francia E, Morcia C, Pasquariello M, Mazzamurro V, Milc JA, Rizza F, Terzi V, Pecchioni N (2016). Copy number variation at the HvCBF4-HvCBF2 genomic segment is a major component of frost resistance in barley. Plant Molecular Biology.

[ref-6] Gehan MA, Park S, Gilmour SJ, An C, Lee CM, Thomashow MF (2015). Natural variation in the C-repeat binding factor cold response pathway correlates with local adaptation of Arabidopsis ecotypes. The Plant Journal.

[ref-7] Ghorbani R, Zakipour Z, Alemzadeh A, Razi H (2020). Genome-wide analysis of AP2/ERF transcription factors family in Brassica napus. Physiology and Molecular Biology of Plants.

[ref-8] Gilmour SJ, Fowler SG, Thomashow MF (2004). Arabidopsis transcriptional activators CBF1, CBF2, and CBF3 have matching functional activities. Plant Molecular Biology.

[ref-9] Gilmour SJ, Zarka DG, Stockinger EJ, Salazar MP, Houghton JM, Thomashow MF (1998). Low temperature regulation of the Arabidopsis CBF family of AP2 transcriptional activators as an early step in cold-induced COR gene expression. The Plant Journal.

[ref-10] Giri J, Dansana PK, Kothari KS, Sharma G, Vij S, Tyagi AK (2013). SAPs as novel regulators of abiotic stress response in plants. Bioessays.

[ref-11] Guan Y, Liu S, Wu W, Hong K, Shi J (2021). Genome-wide identification and cold stress-induced expression analysis of the CBF gene family in Liriodendron chinense. Journal of Forestry Research.

[ref-12] Haake V, Cook D, Riechmann JL, Pineda O, Thomashow MF, Zhang JZ (2002). Transcription factor CBF4 is a regulator of drought adaptation in Arabidopsis. Plant Physiology.

[ref-13] Hao J, Yang J, Dong J, Fei SZ (2017). Characterization of BdCBF genes and genome-wide transcriptome profiling of BdCBF3-dependent and -independent cold stress responses in Brachypodium distachyon. Plant Science.

[ref-14] Hu B, Jin J, Guo AY, Zhang H, Luo J, Gao G (2015). GSDS 2.0: an upgraded gene feature visualization server. Bioinformatics.

[ref-15] Hu Z, Ban Q, Hao J, Zhu X, Cheng Y, Mao J, Lin M, Xia E, Li Y (2020). Genome-wide characterization of the C-repeat binding factor (CBF) gene family involved in the response to abiotic stresses in tea plant (*Camellia sinensis*). Frontiers in Plant Science.

[ref-16] Hwarari D, Guan Y, Ahmad B, Movahedi A, Min T, Hao Z, Lu Y, Chen J, Yang L (2022). ICE-CBF-COR signaling cascade and its regulation in plants responding to cold stress. International Journal of Molecular Sciences.

[ref-17] Jia Y, Ding Y, Shi Y, Zhang X, Gong Z, Yang S (2016). The cbfs triple mutants reveal the essential functions of CBFs in cold acclimation and allow the definition of CBF regulons in Arabidopsis. New Phytologist.

[ref-18] Jiang F, Feng W, Zhen W, Ying L, Shi G, Hu J, Hou X (2011). Components of the arabidopsis CBF cold-response pathway are conserved in non-heading chinese cabbage. Plant Molecular Biology Reporter.

[ref-19] John R, Anjum NA, Sopory SK, Akram NA, Ashraf M (2016). Some key physiological and molecular processes of cold acclimation. Biologia Plantarum.

[ref-20] Jurczyk B, Grzesiak M, Pociecha E, Wlazło M, Rapacz M (2018). Diverse stomatal behaviors mediating photosynthetic acclimation to low temperatures in hordeum vulgare. Frontiers in Plant Science.

[ref-21] Kirschner J, Štěpánek J, Černý T, De Heer P, Van Dijk PJ (2013). Available ex situ germplasm of the potential rubber crop Taraxacum koksaghyz belongs to a poor rubber producer, T. brevicorniculatum (Compositae–Crepidinae). Genetic Resources & Crop Evolution.

[ref-22] Kumar S, Stecher G, Tamura K (2016). MEGA7: molecular evolutionary genetics analysis version 7.0 for bigger datasets. Molecular Biology and Evolution.

[ref-23] Larkin MA, Blackshields G, Brown NP, Chenna R, McGettigan PA, McWilliam H, Valentin F, Wallace IM, Wilm A, Lopez R, Thompson JD, Gibson TJ, Higgins DG (2007). Clustal W and Clustal X version 2.0. Bioinformatics.

[ref-24] Lata C, Prasad M (2011). Role of DREBs in regulation of abiotic stress responses in plants. Journal of Experimental Botany.

[ref-25] Lee CM, Thomashow MF (2012). Photoperiodic regulation of the C-repeat binding factor (CBF) cold acclimation pathway and freezing tolerance in *Arabidopsis thaliana*. Proceedings of the National Academy of Sciences of the United States of America.

[ref-26] Lin T, Xia X, Ruan J, Liu S, Li J (2018). Genome analysis of *Taraxacum kok-saghyz* Rodin provides new insights into rubber biosynthesis. National Science Review.

[ref-27] Lin YH, Hwang SY, Hsu PY, Chiang YC, Huang CL, Wang CN, Lin TP (2008). Molecular population genetics and gene expression analysis of duplicated CBF genes of *Arabidopsis thaliana*. BMC Plant Biology.

[ref-28] Liu Y, Dang P, Liu L, He C (2019). Cold acclimation by the CBF-COR pathway in a changing climate: lessons from *Arabidopsis thaliana*. Plant Cell Reports.

[ref-29] Liu Y, Zhang D, Wang L, Li DQ (2013). Genome-wide analysis of mitogen-activated protein kinase gene family in maize. Plant Molecular Biology Reporter.

[ref-30] Lv K, Li J, Zhao K, Chen S, Nie J, Zhang W, Liu G, Wei H (2020). Overexpression of an AP2/ERF family gene, BpERF13, in birch enhances cold tolerance through upregulating CBF genes and mitigating reactive oxygen species. Plant Science.

[ref-31] Medina J, Bargues M, Terol J, Pérez-Alonso M, Salinas J (1999). The Arabidopsis CBF gene family is composed of three genes encoding AP2 domain-containing proteins whose expression Is regulated by low temperature but not by abscisic acid or dehydration. Plant Physiology.

[ref-32] Mizoi J, Shinozaki K, Yamaguchi-Shinozaki K (2012). AP2/ERF family transcription factors in plant abiotic stress responses. Biochimica Et Biophysica Acta/General Subjects.

[ref-33] Novillo F, Medina J, Salinas J (2007). Arabidopsis CBF1 and CBF3 have a different function than CBF2 in cold acclimation and define different gene classes in the CBF regulon. Proceedings of the National Academy of Sciences of the United States of America.

[ref-34] Park S, Lee CM, Doherty CJ, Gilmour SJ, Kim Y, Thomashow MF (2015). Regulation of the Arabidopsis CBF regulon by a complex low-temperature regulatory network. The Plant Journal.

[ref-35] Park S, Shi A, Mou B (2020). Genome-wide identification and expression analysis of the CBF/DREB1 gene family in lettuce. Scientific Reports.

[ref-36] Potter SC, Luciani A, Eddy SR, Park Y, Lopez R, Finn RD (2018). HMMER web server: 2018 update. Nucleic Acids Research.

[ref-37] Rocco M, Arena S, Renzone G, Scippa GS, Lomaglio T, Verrillo F, Scaloni A, Marra M (2013). Proteomic analysis of temperature stress-responsive proteins in *Arabidopsis thaliana* rosette leaves. Molecular BioSystems.

[ref-38] Schmidt T, Lenders M, Hillebrand A, Van Deenen N, Munt O, Reichelt R, Eisenreich W, Fischer R, Prüfer D, Gronover CS (2010). Characterization of rubber particles and rubber chain elongation in Taraxacum koksaghyz. BMC Biochemistry.

[ref-39] Shen ZJ, Qin YY, Luo MR, Li Z, Ma DN, Wang WH, Zheng HL (2021). Proteome analysis reveals a systematic response of cold-acclimated seedlings of an exotic mangrove plant Sonneratia apetala to chilling stress. Journal of Proteomics.

[ref-40] Shi Y, Ding Y, Yang S (2018). Molecular regulation of CBF signaling in cold acclimation. Trends in Plant Science.

[ref-41] Shu Y, Liu Y, Zhang J, Song L, Guo C (2015). Genome-wide analysis of the AP2/ERF superfamily genes and their responses to abiotic stress in medicago truncatula. Frontiers in Plant Science.

[ref-42] Sun J, Peng X, Fan W, Tang M, Liu J, Shen S (2014). Functional analysis of BpDREB2 gene involved in salt and drought response from a woody plant Broussonetia papyrifera. Gene.

[ref-43] Tang K, Zhao L, Ren Y, Yang S, Zhu JK, Zhao C (2020). The transcription factor ICE1 functions in cold stress response by binding to the promoters of CBF and COR genes. Journal of Integrative Plant Biology.

[ref-44] Thamilarasan SK, Park JI, Jung HJ, Nou IS (2014). Genome-wide analysis of the distribution of AP2/ERF transcription factors reveals duplication and CBFs genes elucidate their potential function in Brassica oleracea. BMC Genomics.

[ref-45] Thomashow MF (1999). PLANT COLD ACCLIMATION: freezing tolerance genes and regulatory mechanisms. Annual Review of Plant Physiology and Plant Molecular Biology.

[ref-46] Thomashow MF (2010). Molecular basis of plant cold acclimation: insights gained from studying the CBF cold response pathway. Plant Physiology.

[ref-47] Wang M, Tan Y, Cai C, Zhang B (2019a). Identification and expression analysis of phosphatidy ethanolamine-binding protein (PEBP) gene family in cotton. Genomics.

[ref-48] Wang P, Chen X, Guo Y, Zheng Y, Yue C, Yang J, Ye N (2019b). Identification of CBF transcription factors in tea plants and a survey of potential CBF target genes under low temperature. International Journal of Molecular Sciences.

[ref-49] Wang Z, Wang F, Tang J, Huang Z, Xiong A, Hou X (2014). C-repeat binding factor gene family identified in non-heading Chinese cabbage is functional in abiotic and biotic stress response but different from that in Arabidopsis. Acta Physiologiae Plantarum.

[ref-50] Xie Q, Ding G, Zhu L, Yu L, Yuan B, Gao X, Wang D, Sun Y, Liu Y, Li H, Wang X (2019). Proteomic landscape of the mature roots in a rubber-producing grass *Taraxacum kok-saghyz*. International Journal of Molecular Sciences.

[ref-51] Yamasaki Y, Randall SK (2016). Functionality of soybean CBF/DREB1 transcription factors. Plant Science.

[ref-52] Zan T, Li L, Xie T, Zhang L, Li X (2020). Genome-wide identification and abiotic stress response patterns of abscisic acid stress ripening protein family members in Triticum aestivum L. Genomics.

[ref-53] Zeng R, Li Z, Shi Y, Fu D, Yin P, Cheng J, Jiang C, Yang S (2021). Natural variation in a type-A response regulator confers maize chilling tolerance. Nature Communications.

[ref-54] Zhang H, Zhu J, Gong Z, Zhu JK (2022). Abiotic stress responses in plants. Nature Reviews Genetics.

[ref-55] Zhao C, Zhang Z, Xie S, Si T, Li Y, Zhu JK (2016). Mutational evidence for the critical role of CBF transcription factors in cold acclimation in Arabidopsis. Plant Physiology.

[ref-56] Zhao K, Zhou Y, Li Y, Zhuo X, Ahmad S, Han Y, Yong X, Zhang Q (2018). Crosstalk of PmCBFs and PmDAMs based on the changes of phytohormones under seasonal cold stress in the stem of Prunus mume. International Journal of Molecular Sciences.

[ref-57] Zhou X, Liao Y, Kim SU, Chen Z, Nie G, Cheng S, Ye J, Xu F (2020). Genome-wide identification and characterization of bHLH family genes from *Ginkgo biloba*. Scientific Reports.

[ref-58] Zhu Z, Ding Y, Zhao J, Nie Y, Zhang Y, Sheng J, Tang X (2016). Effects of postharvest gibberellic acid treatment on chilling tolerance in cold-stored tomato (*Solanum lycopersicum* L.) Fruit. Food and Bioprocess Technology.

