# Supplementary figures and images for: Genome-wide identification of CBF genes and their responses to cold acclimation in Taraxacum kok-saghyz
Source: PeerJ. 2022 May 12;10:e13429. doi: 10.7717/peerj.13429 (PMC9107785; doi:10.7717/peerj.13429)

**Supplemental Figure.1**

**Comparison of CBF motifs between Hevea brasiliensis and A. thaliana**


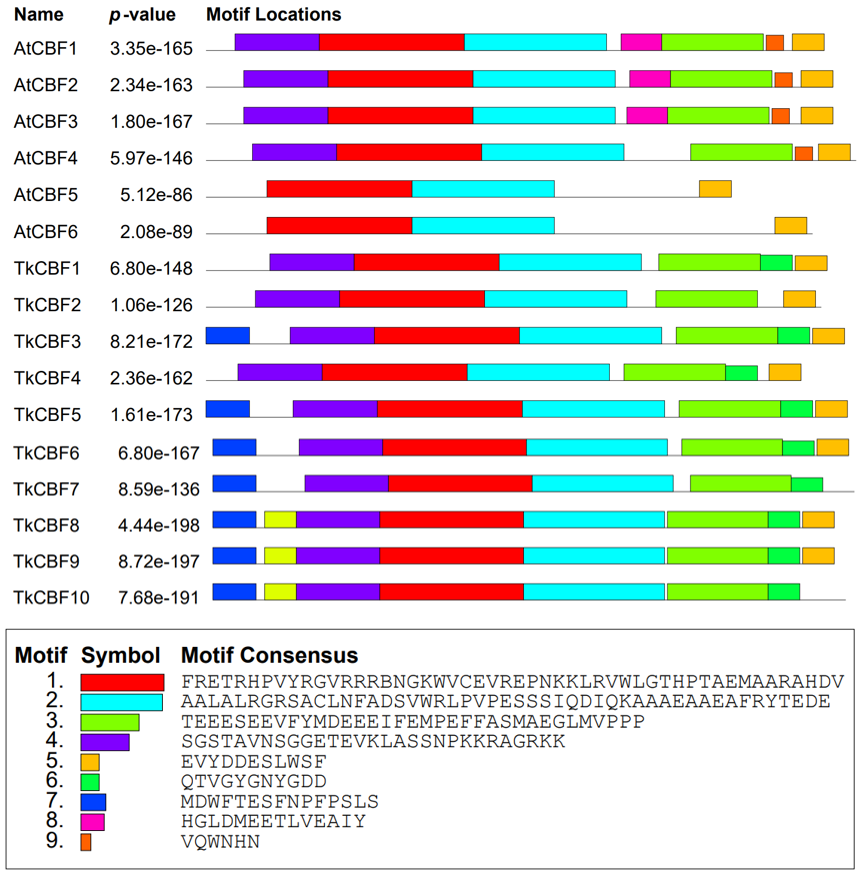

Supplement: Supplemental Information 1 [file peerj-10-13429-s001.docx]

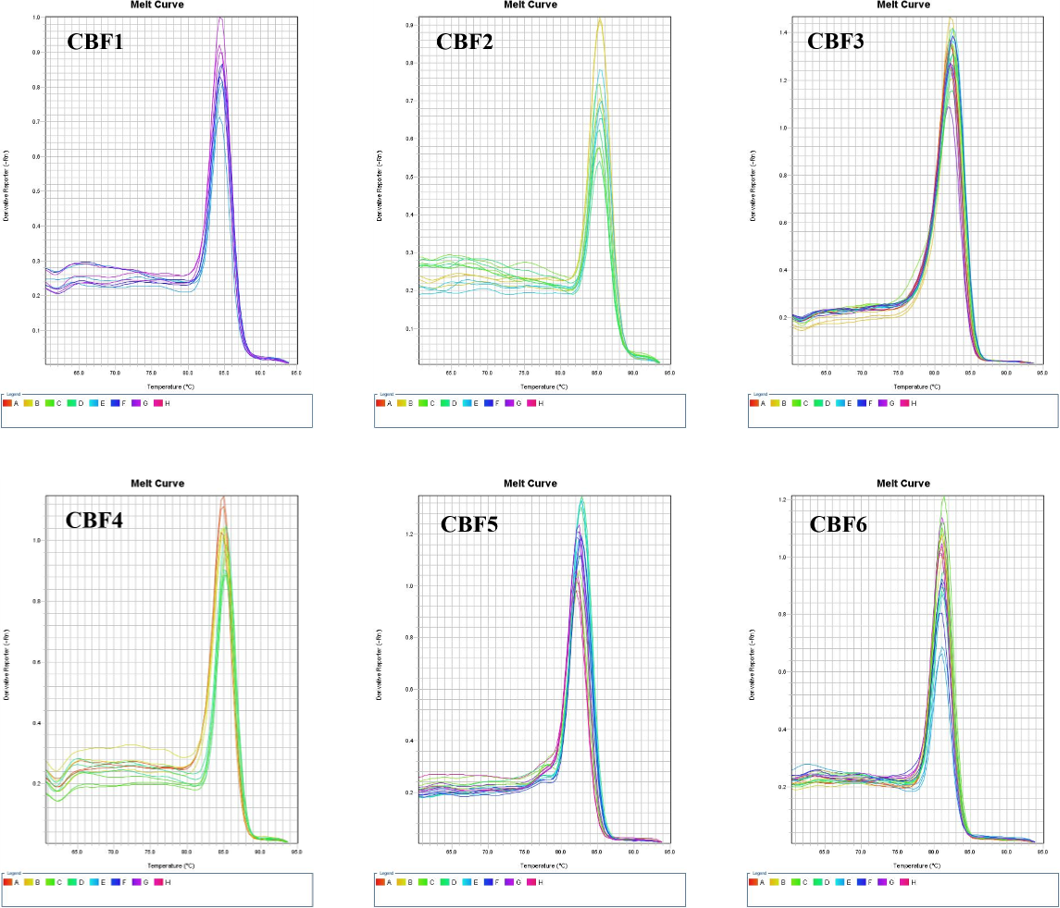

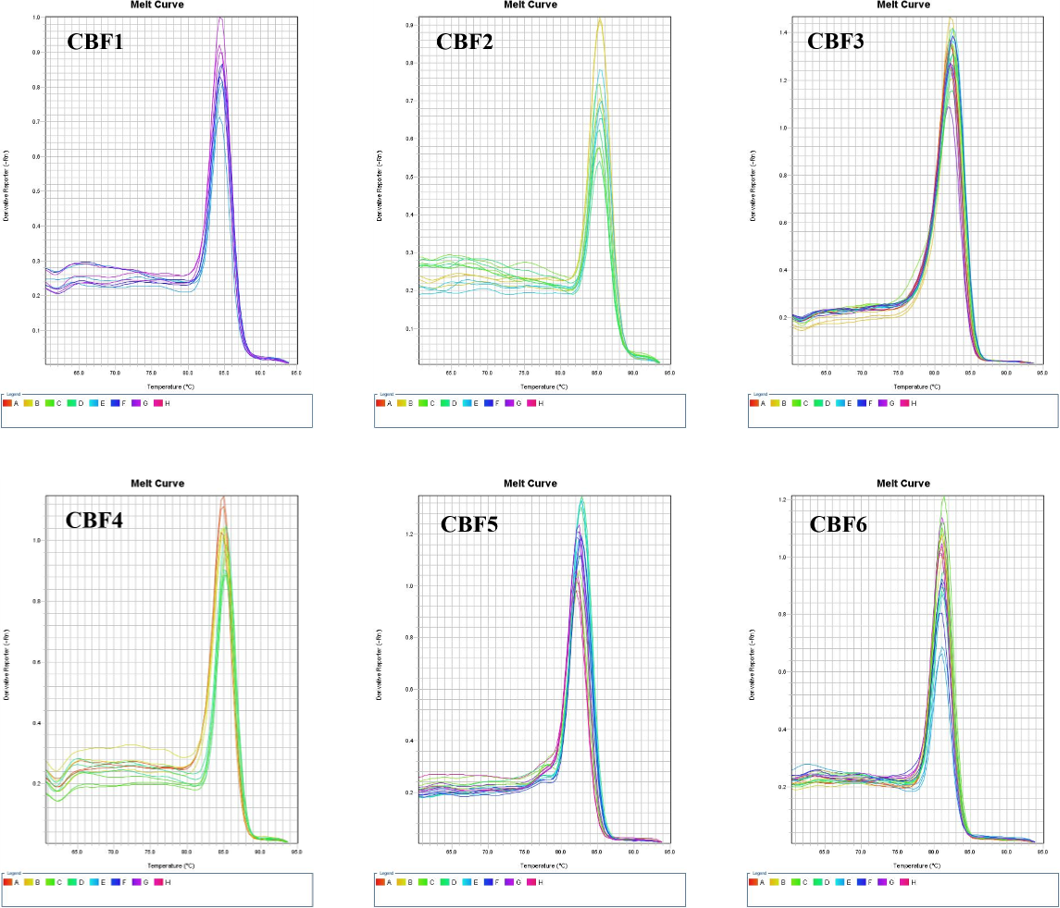


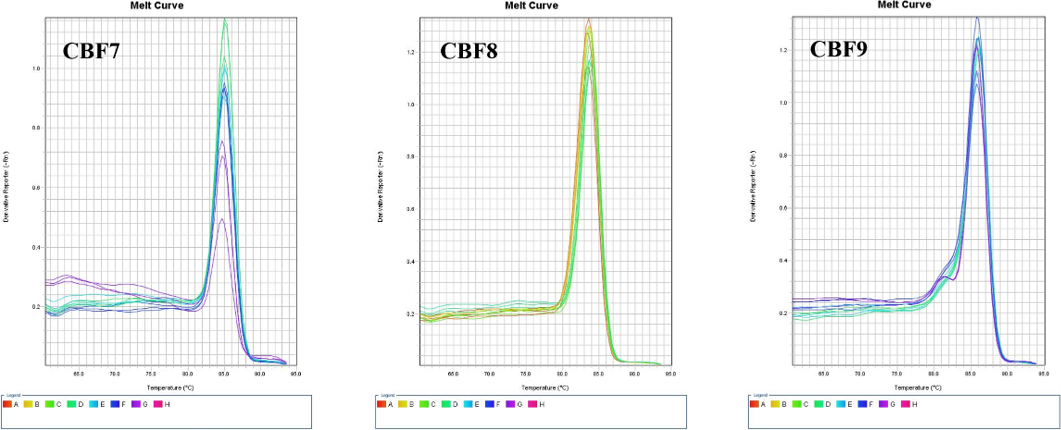


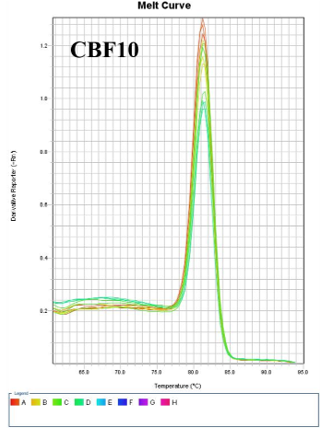

Supplement: Supplemental Information 2 [file peerj-10-13429-s002.docx]
