# Supplementary material for: Genome-wide identification of CBF genes and their responses to cold acclimation in Taraxacum kok-saghyz
Source: PeerJ. 2022 May 12;10:e13429. doi: 10.7717/peerj.13429 (PMC9107785; doi:10.7717/peerj.13429)
Supplement: Supplemental Information 3 [file peerj-10-13429-s003.docx]

Table S1. Primer information of TkCBF family genes

| Gene name Primer sequence(5’-3’) Gene name Primer sequence(5’-3’) | |
| --- | --- |
| TkCBF1 F: GAGACACGCCACCCAGTTTA  R: TAGATACGTCCCCAGCCACA  TkCBF2 F: AGAGGAGTTTGGAATGCCGG  R: CACTTCATCTCCACCGCTGT  TkCBF3 F: CACCATTCTGAATGCGGCTG  R: GTGTTCCTAGCCACACCCTC  TkCBF4 F: TCAGAGTCTGGCTAGGGACC  R: AAACACGCCGATTGACCTCT  TkCBF5 F: ACCTCAAATCGCCGCAAAAC  R: CGCACTTTTTCGGGTTTCGT | TkCBF6 F: GCGGTGCTATCATCGGAAGA  R: TCGTGAAGCCAGCATCACTT  TkCBF7 F: TGGCAATGAGAGGACGATCG  R: CACAATCTCCACCGCATCCT  TkCBF8 F: CCGGGAATTCTGGTGGTGAA  R: GATGCGTCCCTAGCCATACC  TkCBF9 F: GTATGGCTAGGGACGCATCC  R: CCGCTTCTTTGGCAGCTTTT  TkCBF10 F: AGAACTGCCGGGAATTCTGG  R:CGGGTGTCTAGTCTCCCTGA |
