# Supplementary material for: Genome-wide identification of CBF genes and their responses to cold acclimation in Taraxacum kok-saghyz
Source: PeerJ. 2022 May 12;10:e13429. doi: 10.7717/peerj.13429 (PMC9107785; doi:10.7717/peerj.13429)
Supplement: Supplemental Information 4 [file peerj-10-13429-s004.docx]

>GWHTAAAA022478 TkCBF1

ATGACTACTATTATTCGATTCAATACGCCGTACTCCACAGTACAGTCAGCTGAATCTTGTTCAACTTCTGATTGCAGCACCACCGGAAGCTTTTCCGACG

AGGAGGTCATGTTAGCTTCAAAAAACCCCAAGAAGCGAGCTGGGAGGAAGAAGTTCAAAGAGACACGCCACCCAGTTTACCGGGGAGTGCGCCGGAGAAA

TTCCGGCAAATGGGTGTGCGAGGTCAGAGAGCCCAATAAGAAAACCAGGATGTGGCTGGGGACGTATCTAACAGCTGACATGGCTGCCAGAGCTCATGAC

GTGGCGGCTTTGGCTTTGAAGGGGCGATCAGCGTGTTTGAATTTTGCTGACTCCGTGTGGCGGCTGCCGGTCCCGGAATCTAGTAATGTGCAGGACATTA

AGAAGGTGGCGCTTGAGGCGGCTGAGGCTTTTCGGCCGCCGGAGTCTGATGTGGCGGAGGTTGAAGAGAGCAAGGAGTTGACGGTAAATTGTTTGTATAT

GGATGAGGAGGAGATTTTTGGGATGCCGGGGTTTCTTGCCAACATGGCGGAAGGACTGATGCTACCACCACCGATGACCGTTGGATACGACAACTATGAG

GATGACGTGGAATTTAGTTTTGACTCGTCATTGTGGAGTTTCTAA

>GWHTAAAA015650 TkCBF2

ATGGAGATGAACAGTTACAATAATGGGTATTATGATGGATATGTGTACGGGTCTCCGCCGGAGATGGAGGTCGAGGAGTCGTCGGAGGAGTTGATTCTGG

CGGCTAGTAATCCTAAGAAACGTGCCGGGAGGAAGAAGGTTCAGGAGACGAGGCACCCGGTGTTCAGAGGAGTGAGGCAGAGGAGTTCCGGGAAGTGGGT

TTGTGAGGTGAGAGAACCGTATAAGCAGTCGCGGATATGGCTGGGGACTTTTCCGACGGCGGAGATGGCAGCAAGAGCTCATGATGTGGCGGCGTTGGCG

TTGAGGGGGAGGTCGGCTTGTTTGAACTTTGCGGATTCTGCTTGGAGGCTTCCGACTCCGGCGTCATTGGATCCAAACGATATCCGGAAGGCGGCCGTGG

AGGCGGCGGAGGGGTTCCGTGGGGGTGAGGAGGGGGATGGGAGTGGTGGGTCTGGGGAGGGTGTGGGGGTGGGGTACGGGGGTAATGGTGAGTATATGGA

TGAAGAAGAGGAGTTTGGAATGCCGGGGTTGCTTGTGAATATGGCGGAGGGGCTGATGGTACCGCCACCGCCGGCGTACAGCGGTGGAGATGAAGTGGAG

TATGGTGGTGACATATCGTTATGGAGTTACAGTTACTGA

>GWHTAAAA034368 TkCBF3

ATGGACACCTTTATCGAAGCATATAACCCATTTCCAACACTATCACCGGAAAGTGTTCCGTTAGCATACTTTCTGACCTCAAATCGCCGGAAAACCACCA

TTCTGAATGCGGCTGCGGAAGCAGAAGTGAAGCTGGCTTCACGAAACCCGAAAAAGATCGCCGGAAGGAAGAAATTCAGGGAGACTCGACACCCGGTTTA

CAGGGGAGTAAGAATGAGGGATAATGGCAAATGGGTTTGTGAGTTAAGAGAACCAAAGAAGAAATTGAGGGTGTGGCTAGGAACACATCCGACTGCTATA

ATGGCAGCCAGAGCACATGACGTTGCCGCCTTTGCATTCAGAGGGCGATCAGCGTGTTTGAATTTTGCCGACTCCGTGTGGCGGTTGCCGGTTCCCAAAT

CTGGCAAAATAGAAGATATACAAAAGGCAGCTGCAGAAGCGGCGGAGAGTTTTAGATACACGGAGGATGAGACGGAGATTTTGGAAGCAGAGGAGTTGCC

GGAGATTCTGTTTTATTTGGATGAAGAAGATATCTTCGAGATGCCGGAATATTTTGCTAGTATGGCGGAGGGAATGATGGTAGCGCCTCCACAGGCAGTG

AGGTATGGTAGTTACGGAGAAAAGGTGGAAGTTTGTGCCAACGAATCTTTATGGAGCTTCTAG

>GWHTAAAA007906 TkCBF4

ATGGATTCATCAGAAAGTTTTGAGTTTGGATCATCTTCAAACTCCAACTGGAATGCCGCCATTGAAACAGAAATGAAACTAGCTTCACTAACCCCGAAGA

AGAGAGCCGGAAGGAAGAAGTTCCGGGAGACCCGCCACCCGGTTTACCGGGGAGTAAGAATGAGGGATAACGGAAAGTGGGTTTGCGAGGTGAGAGAGCC

AAACACCCAGTTCAGAGTCTGGCTAGGGACCCACCCAACTGCTGAGATGGCAGCCAGGGCACATGACGTGGCAGCTTTGGCGTTCAGAGGTCAATCGGCG

TGTTTGAATTTTGCTGACTCAGTCTGGCGACTGCCGGTTCCTGTATCTAACAGTATACAGGATATACAAAAGGCGGCGGCGGAAGCGGCGGAGGCTTTCA

GACACACGGCGGATGCGATGGAGAATGTGGAAACAAAAGAGTCGCCGGAAGCTCTGTTTTACGAGGACGAGGAGGAGATATTCCAGATGCCGGAGTTTTT

TGCCAGCATGGCGGAAGGACTAATGGTCCCACCACCTGAGTCCGTTGGATATGGCAACAATGGGGAAAATAATCAAAATATGGACTTTTATGCTGACGAG

TGTTTATGGAGTTTTTAG

>GWHTAAAA034735 TkCBF5

ATGGAAGCTTTTGTCGAATCGTCTAACCCATTTCCAGCAACACTATCATCGGAAATTGTTCCGTTGGCATACCTTTTGACCTCAAATCGCCGCAAAACCA

CCATTCTGAATGCGGCAGCGGAAGCGGAAGTGATGCTGGCTTCACGAAACCCGAAAAAGTGCGCCGGAAGGAAGAAATTCAGGGAGACTCGACACCCGAT

TTACAGAGGAGTAAGAATGAGGGATAACGGTAAATGGGTTTGTGAGTTAAGAGAACCGAAGAAGAAGTTGAGGGTGTGGCTAGGGACGCATCCGACTGCT

ATAATGGCAGCCAGAGCACATGACGTTGCCGCCTTTGCATTCAGAGGGCGATCGGCGTGTTTGAATTTTGCCGACTCCGTGTGGCGGTTGCCGGTTCCCA

AATCTGGCAAAATAGAAGATATACAAAAGGCAGCTGCAGAAGCGGCGGAGAGTTTTAGATACACGGAGGATGAGGCGGAGATTTTGGAAACAGAGGAGTT

GCCGGAAATTCTGTTTTATTTGGATGAAGAAGATATTTTCGAGATGCCGGAATATTTTGCCAGCATGGCGGAGGGACTGATGGTAGCGCCTCCGCAAGCA

GTGGGGTATGGCAGTTACGGCGATAAGGTGGAAGTTTGTGATCACGAGTGTTTATGGAGCTTCTAG

>GWHTAAAA034733 TkCBF6

ATGGGTGCCTTTATCGAATCGTCTAACCAATTTCCAGCGGTGCTATCATCGGAAGATGTTTCGTTGACTTACTTTCTTACCTCAAACCGCCGGAAAACCA

CCATTCTGAATGCGGCTGCGGAAGCAGAAGTGATGCTGGCTTCACGAAACCCGAAAAAGAGCGCCGGAAGGAAGAAATTCAGGGAGACTCGACACCCGAT

TTACAGGGGAGTAAGAATGAGGGATAACGGCAAATGGGTTTGTGAGTTAAGAGAACCAAAGAAGAAGTTGAGGGTGTGGCTAGGGACACATCCGACGGCT

ATAATGGCAGCCAGAGCACATGACGTTGCCGCCTTTGCATTCAGAGGGCGATTGGCGTGTTTGAATTTTGCCGACTCCGTGTGGCGGTTGCCGGTTCCCA

AATCTAGCAGAATAGAAGATATACAAAAGGCAGCTGCAGAAGCGGCGGAGAGTTTTAGATACACGGAGGATGAGAGGGAGATTTTGGAAACAGAGGACTT

GCCGGAAATTCTGTTTTATTTGGATGAAGAAGACATTTTAAAGATGCCGGAATATTTTGCCAGCATGGCGGAGGGCCTCATGGTAGCACCTCCGCTGGCG

GTGAGATATGGTAGTTATGGAGATGAGGTGGAAGTTTGTGATGACGAGTGTTTATGGAGTTTCTAA

>GWHTAAAA015648 TkCBF7

ATGGATTATTTTACCGAATCCCATATCCCAATATCTACAGAACTCTCATCGGAAAATACTTCGCTCGAGTCTTCTTCCACTTCCGACTGCAGCAGCACCA

GTATCGCCGGAGGCACTGGTTATTCGGAAGGCGAAGTGATGCTGGCTTCACGAAACCCAAAGAAGAGAGCCGGAAGGAAGAAGGTTAATGAGACTCGCCA

CCCTGTTTTCCGGGGTGTGAGAAGAAGAAGCTCCGGCAAGTGGGTTTGTGAGGTGAGAGTGCCTAATAAGAAAGCACGGGTGTGGCTGGGGACGTATGTC

ACTGCTGAAATGGCGGCGAGGGCACATGACGTGGCGGTGTTGGCAATGAGAGGACGATCGGCGTGTTTAAATTTTGCTGACTCCGTGTGGAGGCTGGCGG

TCCCGGAGTCTAGCAATGTACAGGATATAAAGAGGGCGGCGGCTGAGGCGGCGGAGGCTTTTAAACCCACAGAGGATGCGGTGGAGATTGTGGAAACGAA

GGATTTGGAGGAAGGAGATGTGGTTTTCATGGATGAAGAGGAGATCTTTGCGATGCCGGCGTTTCTTGCCAGCATGGCGGAGGGATTGATGATGCAGCCA

CCTCAGAGGCTTGCGTATGGCAACTCTATGGATAATTTTGAATTTTGTGTTGACGATTTGTGGACTTTTTAG

>GWHTAAAA015647 TkCBF8

ATGGATTGGTTTACAGAATCGTTTAACTCATTTCCATCACTGTCATCAGAAAGTGTTTCCTTTGGGTCGTCTTCCAACTCAAATAACAGCGGAAGCACTG

CCGGGAATTCTGGTGGTGAAACAGAGGTGAAGCTAGCTTCACAAAACCCCAAGAAAAGAGCCGGCCGGAAAAAGTTTAGGGAGACTAGACACCCGGTGTA

CCGGGGAGTGAGGACGAGAGATAACGGCAAGTGGGTTTGTGAGGTGAGAGAGCCAAACACCAAGTTACGGGTATGGCTAGGGACGCATCCCACCGCTGTA

ATGGCGGCAAGGGCACATGACGTGGCTGCTTTGGCGTTCAGAGGGCAATCGGCGTGTTTGAATTTTGCTGACTCGGTGTGGCGACTGCCCATTCCGAACT

CTAGCAGTATACAGGATATACAGAAAGCTGCCAAAGAAGCGGCGGAGGCTTTCAGATACACGGAGGAGACGGTGGAAACAGAGGAGTCGCCGGAAGTTCA

GTTTTATGTTGATGAGGAGATTTTTGAGATGCCGAGGTTTTTTTCCAGCATGGCTGAGGGACTGATGGTCCCACCTCCCCAAACGATGGGGTATGGCAAC

TATGGGGATGATATGGGGTTGTTTTATGATGAGTCTTTATGGAATTTTTAG

>GWHTAAAA010522 TkCBF9

ATGGATTGGTTTACAGAATCGTTTAACTCATTTCCATCACTGTCATCAGAAAGTGTTTCCTTTGGGTCGTCTTCCAACTCAAATAACAGCGGAAGCACTG

CCAGGAATTCTGGTGGTGAAACAGAGGTGAAGCTAGCTTCACAAAACCCCAAGAAAAGAGCCGGCCGGAAAAAGTTTAGGGAGACTAGACACCCGGTGTA

CCGGGGAGTGAGGACGAGAGATAACGGCAAGTGGGTTTGTGAGGTGAGAGAGCCAAACACCAAGTTACGGGTATGGCTAGGGACGCATCCCACCGCTGTA

ATGGCGGCAAGGGCACATGACGTGGCTGCTTTGGCGTTCAGAGGGCAATCGGCGTGTTTGAATTTTGCTGACTCGGTGTGGCGATTGCCCATTCCGAACT

CTAGCAGTATACAGGATATACAAAAAGCTGCCAAAGAAGCGGCGGAGGCTTTCAGATACACCGAGGAGACGGTGGAAACAGAGGAGTCGCCGGAAGTTCA

GTTTTATGTTGATGAGGAGATTTTTGAGATGCCGAGGTTTTTTTCCAGCATGGCTGAGGGACTGATGGTCCCACCTCCCCAAACGATGGGGTATGGCAAC

TATGGGGATGATATGGGGTTGTTTTATGATGAGTCTTTATGGAATTTTTAG

>GWHTAAAA010523 TkCBF10

ATGGATTGGTTTACAGAATCGTTTAACTCATTTCCATCACTGTCATCAGAAAGTGTTTCCTTTGGGTCGTCTTCCAACTCAAATAACAGCGGAAGAACTG

CCGGGAATTCTGGTGGTGAAACAGAGGTGAAGCTAGCTTCACAAAACCCCAAGAAAAGAGCCGGCCGGAAGAAGTTCAGGGAGACTAGACACCCGGTGTA

CCGGGGAGTGAGGACGAGAGATAACGGCAAGTGGGTTTGTGAGGTGAGAGAGCCAAACACCAAGTTACGGGTATGGCTAGGGACGCATCCCACCGCTGTA

ATGGCGGCAAGGGCACATGACGTGGCTGCTTTGGCGTTCAGAGGGCAATCGGCGTGTTTGAATTTTGCTGACTCGGTGTGGCGACTGCCCATTCCGAACT

CTAGCAGTATACAGGATATACAGAAAGCTGCCAAAGAAGCGGCGGAGGCTTTCAGATACACGGAGGAGACGGTGGAAACAGAGGAGTCGCCGGAAGTTCA

GTTTTATGTTGATGAGGAGATTTTTGAGATGCCGAGGTTTTTTTCCAGCATGGCAGAGGGACTGATGGTCCCACCTCCCCAAACGATGGGGTATGGCAAC

TATGGGGATGATATGGGGTTGAGTGCCTTTTCTTCTCATCATTATGGGAAATTACCTCAATAA
